# The Causes of Success and Failure

**Published:** 1915-07-03

**Authors:** W. A. Potts

**Affiliations:** Medical Investigator Royal Commission on the Feeble-Minded.


### MENTAL TESTS IN CHILDHOOD.

### The Causes of Success and Failure.

By W. A. POTTS, M.D. Edin., Medical Investigator Royal Commission on the Feeble-Minded.

Dr. W. A. Potts contributed a paper entitled "What tests in childhood are best calculated to throw light upon their capacities for future work—*i.e.* for earning their own living?" Dr. Potts said: If there is anyone who has come here to-day hoping that I am going to reveal a *précis* of simple tests, by the use of which a careful investigator can decide whether a defective will develop into a wage-earner, I am afraid that person will be disappointed. Those of you who have considered the problem I am approaching know how complex and difficult it is; you know also that the whole project of mental tests is only in its infancy, and that the particular science which seeks to correlate ability before the age of sixteen with wage-earning capacity later is non-existent.

Under these circumstances it might appear that a paper with this title would be of little use. I myself, however, and others also, have refused to certify under the Mental Deficiency Act some "defectives" on the ground that they would ultimately be able to support themselves. There are several points of view from which to approach my problem; the most satisfactory decision would be attained by taking a large group who were submitted to many tests ten years ago, and inquiring what tests those now supporting themselves passed, and in what directions the unsuccessful failed. This information, however, is not forthcoming. We are driven, therefore, to other methods, such as considering what are the causes of success and failure in the careers of normal individuals, and then, if we believe that they will operate also in the case of defectives, devising a set of tests to determine them. We may also take a group of defectives over twenty years of age, earning wages, and find what good qualities they have in common, comparing them with the group who are unemployed.

As regards the causes of success and failure in life, the former, especially if it is to be marked, depends on the possession of four principal qualities: (1) Ability; (2) strength of character and will-power, in which as a subsidiary should be included ambition; (3) good health; (4) pluck. Moderate ability of certain kinds is by no means lacking in the feeble-minded; what particular form it should take I will discuss in a moment. The other characteristics enumerated are just as essential for success for "defectives." Most "defectives" are lacking in ambition, many see no reason why they should earn their own living; those who are avowedly anxious to support themselves or to qualify as a soldier or a cook have a driving power of real value. Good health is not always ranked amongst the essentials for success; I may be reminded that world-famous work has been accomplished by the victims of serious disease. This is true, but it is also true that this has only been done by the help of such ability or determination as is not likely to be found among "defectives." This consideration leads to the important reflection that mental defect is in itself such a serious impediment that the unfortunate victim can afford to carry no other handicap.

For this reason a useful decision as to prospects in after-life can only be given by a medical man who is also a psychologist, after examining the child to make sure there is no defect of vision or hearing, no other defect of consequence, and no other active or latent disease. The medical investigator's final opinion cannot be given without a thorough knowledge of the family history and

**Conference** (*continued*).

record, particularly in regard to the presence and frequency of consumption, insanity, epilepsy, asthma, and heart disease, and the ages at which they developed in previous generations.

In order to emphasise the value of good physical condition I may tell you that of the first seventy-nine cases brought up to be dealt with under the Mental Deficiency Act that I examined—these seventy-nine, of course, all being failures—thirty-eight, or nearly 50 per cent., were suffering from acute disease or some serious physical disability. This is confirmed by an investigation, to which I shall refer directly, into the careers of sixty-eight defectives, eighteen of whom were out of work. Half of those out of work were suffering from disease or serious defect.

The last essential enumerated for success is pluck, a virtue the necessity for which is not always realised. Careful analysis will show, however, that many tragedies are due to lack of this quality. The man who is bound to win is not overwhelmed when an unexpected calamity overtakes him; still less is he haunted by those vague fears of impending disaster which cause so much distress to some weaklings, and which are particularly common among the feeble-minded. Courage is especially necessary for the feeble-minded, who otherwise furnish a ready butt for rough companions. I know a number of defectives who will not go to work because their mates play tricks on them or make fun of them. One of these is a skilled musician, fully capable of work in a music warehouse.

On the other hand, one of the most powerful defectives I have known was always free from annoyance of this kind; he was very powerful, and his companions stood in great awe of him. Luck comes in. A schoolmistress told me that one defective earning wages was very stupid, and never would have earned anything unless he had had a very good mother, who was determined he should do something, and never rested until she had got him the one situation he was fit for, a post as an errand-boy. The feeble-minded are frequently unduly susceptible to extraneous influences: a kind master and a sympathetic teacher count for much more than with the normal.

On studying the records of sixty-eight defectives over eighteen years of age for reading, writing, arithmetic, and manual work it is obvious that aptitude for manual work is essential.

Dividing these attainments at school into three classes, I find that of those now at work 72 per cent. were first-class at manual work, while of those not at work only 33 per cent. were good at it. As regards other school subjects, reading does not seem to be of much use as a guide. Paradoxical as it sounds, if you are defective it may be better not to be able to read at all, because you may be a pure word-blind case, not defective all through, and may be very good at everything else and capable of earning good wages.

Arithmetic gives a better clue. Of those at work 55 per cent. were first-class at arithmetic.

Writing is also some guide, at any rate in a negative way; it is so much easier to teach a defective to write than to read or calculate that he must be of low grade if he does not learn it at all. Really, writing may be looked upon as a form of manual work; some defectives can copy beautifully, but cannot read what they have written. It is only in rare instances that all calculations are upset. One surprise is a youth who was very bad at school in every way, but is now in the Army serving as a dispatch-rider at the Front. Many cases are now in the Army, and more than one is doing well; in the Army there is the strict discipline and constant supervision defectives require.

